# Analysis of 180 Genetic Variants in a New Interactive FX Variant Database Reveals Novel Insights into FX Deficiency

**DOI:** 10.1055/a-1704-0841

**Published:** 2021-11-23

**Authors:** Victoria A. Harris, Weining Lin, Stephen J. Perkins

**Affiliations:** 1 Research Department of Structural and Molecular Biology, University College London, Darwin Building, Gower Street, London, United Kingdom

**Keywords:** coagulation factors, hemostasis, protein structure/folding, gene mutations, inherited coagulation disorders

## Abstract

Coagulation factor X (FX), often termed as Stuart–Prower factor, is a plasma glycoprotein composed of the γ-carboxyglutamic acid (GLA) domain, two epidermal growth factor domains (EGF-1 and EGF-2), and the serine protease (SP) domain. FX plays a pivotal role in the coagulation cascade, activating thrombin to promote platelet plug formation and prevent excess blood loss. Genetic variants in FX disrupt coagulation and lead to FX or Stuart–Prower factor deficiency. To better understand the relationship between FX deficiency and disease severity, an interactive FX variant database has been set up at *https://www.factorx-db.org* , based on earlier web sites for the factor-XI and -IX coagulation proteins. To date (April 2021), we report 427 case reports on FX deficiency corresponding to 180 distinct *F10* genetic variants. Of these, 149 are point variants (of which 128 are missense), 22 are deletions, 3 are insertions, and 6 are polymorphisms. FX variants are phenotypically classified as being type I or II. Type-I variants involve the simultaneous reduction of FX coagulant activity (FX:C) and FX antigen levels (FX:Ag), whereas type-II variants involve a reduction in FX:C with normal FX:Ag plasma levels. Both types of variants were distributed throughout the FXa protein structure. Analyses based on residue surface accessibilities showed the most damaging variants to occur at residues with low accessibilities. The interactive FX web database provides a novel easy-to-use resource for clinicians and scientists to improve the understanding of FX deficiency. Guidelines are provided for clinicians who wish to use the database for diagnostic purposes.

## Introduction

**Fig. 1:**
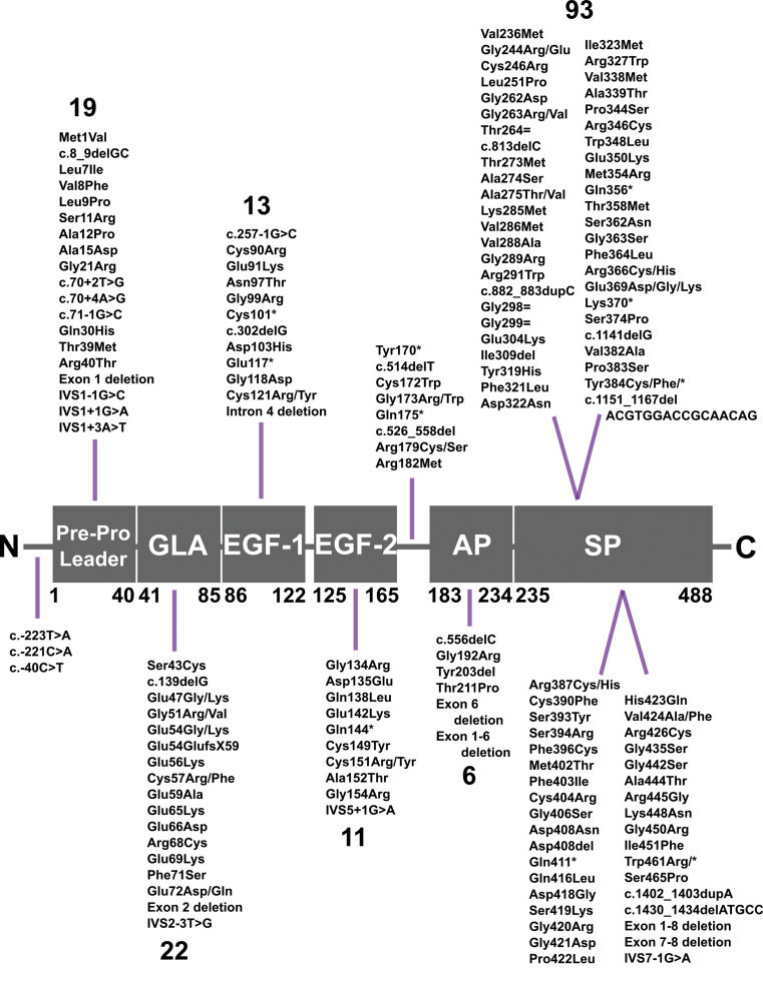
Distribution of the 180 variants identified within the *F10* gene. The six protein segments are not drawn to scale. The number of variants in each of the respective domains is shown in large font above or below the variant lists. Intronic variants are included in their respective domains according to sequence numbering. The residue numbering under the domains marks the amino acids framing each domain, given in HGVS format. N and C represent the N- and C-termini, respectively, of factor X (FX). Only 177 variants are displayed in this schematic as the remaining variants are undefined insertions and deletions. AP, activation peptide; EGF, epidermal growth factor; GLA, γ-carboxyglutamic acid; SP, serine protease.

Factor X (FX) is a vitamin-K-dependent serine protease encoded by the *F10* gene, located on chromosome 13. *F10* is composed of eight exons and is 27 kb in length. The gene encodes a prepeptide and propeptide, cumulatively referred to as the pre–pro leader, a γ-carboxyglutamic acid (GLA) domain, two epidermal growth factor domains (EGF-1 and EGF-2), a 52-amino acid activation peptide (AP), and a catalytically active serine protease (SP) domain ( Fig. 1 ). 1 In the coagulation cascade, FX is responsible for the conversion of prothrombin to thrombin to drive the formation of the platelet plug. FX is synthesized as a zymogen and is activated via the extrinsic or intrinsic coagulation pathways, involving the tissue factor (TF)/factor VIIa (FVIIa) complex or the factor VIIIa (FVIIIa)/factor IXa (FIXa) complex, respectively. 2 Activation involves cleavage of the Arg234-Ile235 bond in the FX heavy chain, releasing the 52-amino acid AP and activated FXa ( Fig. 1 ). Ile235 then forms a salt bridge with Asp418 in the substrate binding pocket. Additional FX cleavage at Lys475-Ser476 is often observed but has no major functional implications. 3 FXa typically complexes with cofactor FVa to form the prothrombinase complex, enhancing prothrombin affinity, and catalysis by 10 ^5^ -fold. This complex interacts with membranous-phosphatidylserine in a Ca ^2+^ dependent manner. Following membrane assembly, prothrombinase cleaves prothrombin at two distinct sites, Arg271 and Arg320, yielding the N-terminal peptide and disulphide linked thrombin. Thrombin subsequently promotes clot formation. 4 5 In addition to playing a direct role in the coagulation cascade, FXa is implicated in conditions such as tissue fibrosis, atherosclerosis, and angiogenesis. 6 FXa binds the protease-activated receptors, PAR-1 and PAR-2, in vascular, immune, and epithelial cells. FXa cleaves the N-termini of PAR-1 and PAR-2, creating a novel N-terminus that functions as a tethered ligand that leads to the triggering of intracellular signaling cascades. 1 7

Experimentation involving *F10* KO mice emphasizes the importance of FX in physiological function. The absence of FX results in partial embryonic lethality and fatal neonatal bleeding. 1 Similarly, in humans, variants that impede FX activity lead to disease states, commonly termed as congenital FX deficiency. Such states can also be referred to as Stuart–Prower factor deficiency, named after the first recognized deficient patients. 8 9 FX deficiency occurs at a frequency of 1:500,000 to 1:1,000,000 across the globe, with 1 in 500 individuals believed to be heterozygous carriers. 9 10 FX deficiency is more prevalent in regions where consanguineous marriage remains common, such as in the Middle East. 9 11 12 Deficient patients experience variable bleeding symptoms, ranging from easy bruising, gum bleeding, nose bleeds and hematuria in mild cases to hemarthrosis, hematomas, intramuscular bleeds, gastrointestinal tract bleeds, and intracranial hemorrhage in more severe cases. All FX deficient patients are at greater risk of prolonged postoperative bleeding. 8 9 10 11 12 13 The predominant treatments for FX deficiency include FX replacement and the use of antifibrinolytics. 13 14 15 16 Antifibrinolytics such as ε-aminocaproic and tranexamic acid or fibrin glue are often used for the treatment of minor bleeds. 15 16 17 Variants causing FX deficiency are characterized as having cross-reacting material negative or positive (CRM ^−^ or CRM ^+^ ) phenotypes. CRM ^−^ or “type I” variants are those that result in the parallel reduction of FX coagulant activity (FX:C) and FX plasma antigen levels (FX:Ag). Contrastingly, CRM ^+^ or “type II” variants reduce FX:C without impacting the FX:Ag level. Such variants are functional variants that go undetected by cellular quality control mechanisms. 8 13 FX:C in healthy human plasma ranges from 50 to 150 IU/dL. 18 Mildly deficient patients have 6 to 10% of normal FX:C, moderately deficient patients 1 to 5% and severely deficient patients <1%. 9 14 16

The diagnosis and treatment of FX deficiency will benefit from a more complete understanding of the correlation between the *F10* genotype and the bleeding severity. 19 To better understand this relationship, we have developed the first interactive database at *https://www.factorx-db.org* for FX variants. The database contains genetic information from literature sources in relation to 180 unique variants from 427 patients, as well as structural information from FXa protein crystallography. The new FX database was adapted from our previously developed interactive coagulation factor databases for factor XI (FXI) and factor IX (FIX) at *https://www.factorxi.org* and *https://www.factorix.org* which underwent initial development in 2003 and 2013, respectively. 20 21 22 In this new FX database, we show how the extended domain structure of FXa is correlated with the distribution of types I and II variants where the phenotype is reported. We compare this distribution with that in FXI when our recently updated variant and structural analyses indicate a predominance of type-I variants in FXI. 23 We show that changes in the solvent surface accessibility of individual residues are well correlated with the occurrence of genetic variants in the FX structure, regardless of the phenotypic classification. We offer guidelines on the interpretation of deleterious variants in the database. The availability of this new database will help clarify the molecular basis of FX deficiency for clinicians and scientists.

## Methods

### Source of the Factor-X Database

The new interactive FX web database at *https://www.factorx-db.org* carries 180 distinct *F10* genetic variants that are associated with FX deficiency. The database was created at University College London and the web site copyright is retained by S. J. Perkins and University College London. Database copying is not permitted without explicit permission. The FX database was derived from the newly upgraded FXI database at *https://www.factorxi.org* , and the FIX database at *https://www.factorix.org* . 20 21 22 The variants in the FX database were populated through online literature searches of peer-reviewed articles, primarily those found on PubMed at *https://pubmed.ncbi.nlm.nih.gov/* . The literature cut-off date was April 2021 from which a total of 180 unique FX variants and 427 patients were extracted. The variants were compiled into a spreadsheet to establish the FX MySQL database, using phpMyAdmin software ( *https://www.phpmyadmin.net/* ) as an intermediary platform. The database is maintained on the University College London server and utilizes HTML programming, PHP, and JavaScript to enable public access via the web. If desired for personal or private research use only, a list of the FX variants and their associated fields can be downloaded from the variants' menu on the web site ( Supplementary Materials S1 and S2 ).

### Analysis of Factor-X Variants

The FX database records DNA changes in Human Genome Variation Society (HGVS) format, where +1 refers to the A of the ATG initiation codon, at the start of the 40-residue pre–pro leader peptide. When publications have used legacy numbering for DNA changes, the origin of the residue numbering is stated clearly in the variant comments section on the web site. Protein changes are recorded in HGVS format, with codon +1 referring to the ATG initiation codon. To facilitate comparison with older publications, legacy numbering is presented also for protein changes on the database, with codon +1 referring to the first codon of the mature unactivated FX protein (+41 in HGVS numbering).

While 136 FXa crystal structures are available on the PDB database ( *https://www.rcsb.org* ), many structures do not show the full-length FXa protein, with many lacking the GLA and EGF-1 domains entirely. Of the 136 FXa structures, PDB ID: 1P0S was alone in successfully determining the GLA domain, yet this crystal structure did not model the EGF-1 domain. 24 Five FXa crystal structures successfully determined the EGF-1 domain, with PDB ID: 1XKA encompassing the most light- and heavy-chain residues at the highest resolution, showing the EGF-1, EGF-2, and SP domains in full. 25 The 1P0S crystal structure showed a structural resolution of 0.280 nm and a goodness-of-fit *R* -value of 0.207. 24 The 1XKA model showed a higher resolution of 0.230 nm and an improved *R* -value of 0.196. 25 Thus, the 1P0S structure was superimposed in full onto the 1XKA structure to result in a full-length merged FXa model with all four domains visible in an extended conformation. The 1P0S structure was then edited out, leaving only its GLA domain along with the full 1XKA structural model. The complete merged FXa model consisted of a light chain ranging from Ser43 to Arg179 and a heavy chain ranging from Ile235 to Arg469 ( Fig. 1 ). The 1XKA structure was retained as much as possible owing to its improved resolution compared with 1P0S. The Ramachandran analysis at *http://molprobity.biochem.duke.edu* was used to confirm the accuracy of the model. 26 Our final native FXa model was not refined by energy minimization nor was the structure refined after making the amino acid substitutions.

To interpret the effect of the FX missense variants, four different substitution analyses were performed on them, namely, Polymorphism Phenotyping v2 (PolyPhen-2) at *http://genetics.bwh.harvard.edu/pph2/* , the Sorting Intolerant From Tolerant (SIFT) at *https://sift.bii.a-star.edu.sg/www/SIFT_seq_submit2.html* , the Protein Variation Effect Analyzer (PROVEAN) at *http://provean.jcvi.org/seq_submit.php* , and the Grantham analysis, using the Grantham matrix proposed in 1974. 27 28 29 30 Both the PolyPhen-2 and SIFT algorithms give variants scores ranging from 0.0 to 1.0. However, in the PolyPhen-2 algorithm, the closer to 1.0, the prediction score is, the more damaging the variant is likely to be. The SIFT scores are reversed where values closer to 0.0 predict more damaging variants and those closer to 1.0 are more tolerated. The PROVEAN threshold used was −2.5, thus variants with scores below the threshold were considered deleterious and those above −2.5 were neutral. The Grantham analysis differs from the other three in that it is not sequence specific and is purely based on the amino acids undergoing substitution. The Grantham scores range from 0 (silent variants) to 215, with larger scores indicating more radical and likely more damaging substitutions.

The Definition of Secondary Structure of Proteins (DSSP) tool at *https://www3.cmbi.umcn.nl/xssp/* was used to identify the secondary structure of each residue in the merged FXa structure. 31 32 DSSP was applied both to the merged FXa structure, as well as to the four separate FXa domains. Residues were individually assigned secondary structures to be one of H (α-helix), B (β-bridge), E (extended β-strand), G (3 _10_ helix), I (π-helix), T (hydrogen-bonded turn), S (bend), or C (undefined coil region). DSSP was also used to determine the relative surface accessibility of each residue in the FXa crystal structure in Å ^2^ . The accessibilities were converted into percentage accessibility by dividing the DSSP output by the theoretical solvent accessible surface area of the amino acid sidechain in question. 33 The results were simplified as follows. Percentage accessibilities of 0 to 9% were given the value 0, 10 to 19% the value 1, 20 to 29% the value 2, and so on. Residues with accessibilities of 0 or 1 were classified as buried and those with accessibilities of 2 to 9 were classified as solvent exposed.

## Results

### The New Factor X Interactive Web Database

**Fig. 2:**
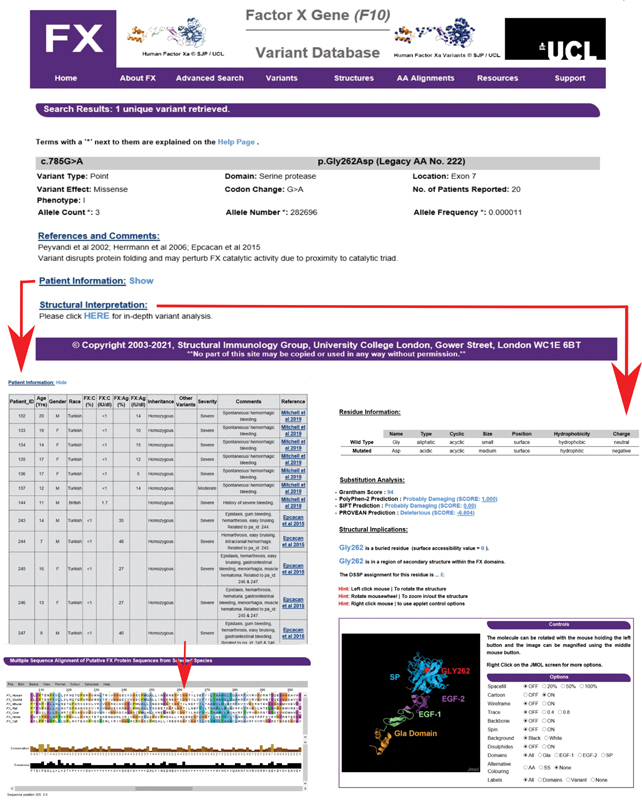
Screenshots of the new factor X (FX) interactive web database to illustrate the genetic and structural analysis made for the Gly262Asp variant. The upper panel displays the output when residue 262 is inputted on the FX web site home page. By clicking “Show” next to the patient information heading, the lower left panel lists genetic information for the 20 patients reported with the Gly262Asp variant, of which the first 12 records are visible here, together with the source of the patient record. The multiple sequence alignment is shown underneath with Gly262 highlighted by a red arrow. Clicking “HERE” under the structural interpretation heading gives the image shown on the bottom right panel. This assesses the buried or exposed accessibility of the variant and its location in the FXa protein structure. Four different substitution analyses (the Grantham, PolyPhen-2, the Sorting Intolerant From Tolerant [SIFT], and PROVEAN) are provided to help predict the likely effects of each missense variant on FXa. A JMol view of the FXa structure is displayed that can be rotated and zoomed into as desired. Additional information regarding the amino acid side chain properties of both the wild type and mutant residues is also displayed on this page.

The interactive FX database ( *https://www.factorx-db.org* ) presents genetic and structural information in relation to 180 FX variants and 427 patients, sourced from 118 research articles ( Fig. 1 ). Specific genetic features in the database include basic and advanced search tools, an interactive clickable variant map, and interspecies *F10* multiple sequence alignments, permitting phylogenetic analysis of the *F10* gene (left, Fig. 2 ). Additionally, the database offers structural analyses of the identified variants, a list of published FX crystal structures, and links to further FX resources (right, Fig. 2 ). These features are accessed from the home page which features movies of FXa with and without its associated variants. From the home page, the basic search tool enables users to search for variants of specific type or with defined cDNA or amino acid numbers. The advanced search tool allows for more explicit searches, including options to search by variant effect, location, and/or reference. The genetic searches report on variant information, patient information, and relevant comments. Variant information includes variant type, effect, location, and allelic specific information. Allelic frequencies (AFs) are provided for variants wherever possible using data supplied by the genome aggregation database (gnomAD) version 2.1.1 at *https://gnomad.broadinstitute.org/* . 34 The gnomAD v2.1.1 dataset spans 125,748 exome sequences and 15,708 whole-genome sequences and contains 51 (28%) of the 180 identified FX variants. The AF is used as an indication of the relative frequency of a given variant at a specific genetic locus in a genome. The AF cut-off used was 0.01, thus those AF > 0.01 indicated a common variant. Of the 51 variants present in the gnomAD dataset, only two had AF > 0.01, both of which corresponded to known polymorphisms (Thr264Thr and c.-40C > T). The predominance of rare FX variants in the gnomAD population highlights the importance of FX gene conservation. The anonymized patient information reports on patient age, gender, race, assay results, and disease severity.

The database provides structural analyses for the missense variants which can be mapped onto an interactive FXa structure for inspection. A molecular JMol viewer for each variant is provided through Java software. It should be noted that Java applets need to be enabled within the browser to permit these features to be seen. Users are able to apply prediction software to better understand the structural effects of any potential missense variant (right, Fig. 2 ). Users have the option to submit novel variants and to contact the site administrator with additional queries. The database site map and help pages make for easy navigation and comprehension of the site.

### Classification of Factor X Variants and Polymorphisms

**Fig. 3:**
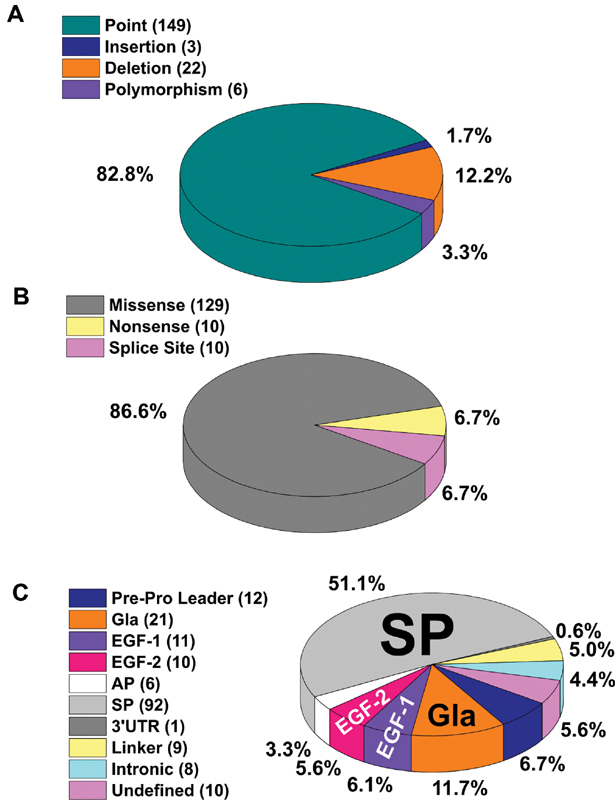
Distribution of the 180 variants identified within the *F10* gene. The panels ( **A** – **C** ) illustrate the breakdown of the 180 factor X (FX) variants by variant type, effect and location within the *F10* gene sequence. The charts illustrated here distinguish the nondisease associated polymorphisms from the disease-causing genetic variants. ( **A** ) Relative frequency of four different types of genetic variants in the *F10* gene; ( **B** ) Genetic classification of 149 point variants in the *F10* gene; ( **C** ) Relative distribution of the 180 FX variants across the *F10* gene and within the protein domains. The six undefined variants include two unique deletions that stretch across exons 1–6 and 1–8, respectively, one undefined deletion and three missense variants preceding the *F10* coding sequence. AP, activation peptide; EGF, epidermal growth factor; GLA, γ-carboxyglutamic acid; SP, serine protease.

**Fig. 4:**
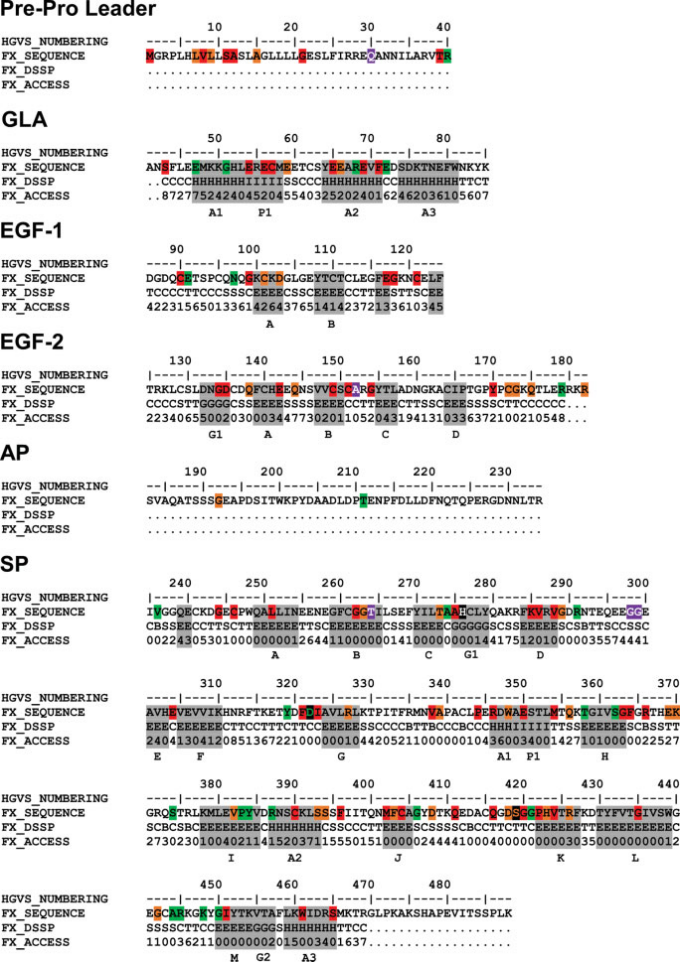
Secondary structure and accessibility analysis of variants in the merged FXa crystal structure. The factor X (FX) sequence is shown with the major secondary structure assignments highlighted in gray boxes. Residues are denoted either H (α-helix), B (β-bridge), E (extended β-strand), G (3 _10_ helix), I (π-helix), T (hydrogen-bonded turn), S (bend), or C (undefined coil region). The SP catalytic triad His276-Asp322-Ser419 is highlighted in black. β-strands are labeled alphabetically in the order in which they occur. The β-strands in the EGF-1, EGF-2 and SP domains are denoted A-B, A-D, and A-M respectively. The α-helices are denoted A1-A3, 3 _10_ -helices G1-G2, and π-helices P1. The positions of 136 point variants found in the *F10* gene are highlighted in boxes, where 57 red boxes denote type-I mutations, 26 green boxes denote type-II mutations, and 33 orange boxes denote mutations with unknown phenotype. Several boxes correspond to multiple variants at one residue position. The five purple boxes denote nondisease associated polymorphisms. All numbering is in HGVS format. AP, activation peptide; EGF, epidermal growth factor; GLA, γ-carboxyglutamic acid; SP, serine protease.

Of the 180 variants presented in the FX database, 82.78% are point variants, 12.22% deletions, 3.33% polymorphisms, and 1.67% insertions ( Fig. 3A ). The 149 point variants are further subdivided into missense (86.58%), nonsense (6.71%), and splice site (6.71%) variants ( Fig. 3B ). Of the 180 genetic variants, 174 are disease-associated alterations and six are nondisease associated polymorphisms, including the above pair (Thr264Thr and c.-40C > T). The 180 variants are spread across the *F10* gene, with 51.11% clustered in the large SP domain ( Fig. 3C ). As illustrated in Fig. 4 , 46.55% of the disease-causing variants are phenotypically classified as type I, 16.09% as type II, and 37.36% have an unknown phenotype. Here, type-II variants are characterized by a FX:C to FX:Ag ratio of <0.7. Closer phenotypic analysis revealed that 64.29% of the type-II variants were in the SP domain, while 14.29% occurred in the GLA domain, 7.14% in the EGF-1 domain, 7.14% in the EGF-2 linker region, and less than 5.00% in each of the pre–pro leader and AP sequences. The abundance of type-II variants in the SP and GLA domains highlights their functional activity in terms of peptide hydrolysis and in binding to host cell membranes respectively. The reduced abundance of type-II variants in the EGF-1 and EGF-2 domains and the abundance of type-I variants in these indicates their importance in the formation of a correctly-folded protein structure. Of the 10 most commonly occurring FX variants in patients (Pro383Ser, 25 occurrences; Gly21Arg, 21; Gly262Asp, 20; Glu54Lys, 16; Glu91Lys, 15; Asp322Asn, 15; Ser374Pro, 14; Gly406Ser, 14; Gly421Asp, 13; Glu142Lys, 12; in order of frequency as shown), six are type II and four are type I. Interestingly, the Asp322Asn variant is a type-II variant involving the catalytic triad residue Asp322, meaning that this variant loses FXa catalytic activity. The occurrence of this disease-causing variant (15 case reports to date) highlights the importance of the FX catalytic triad in FXa proteolytic function. 35 36

**Fig. 5:**
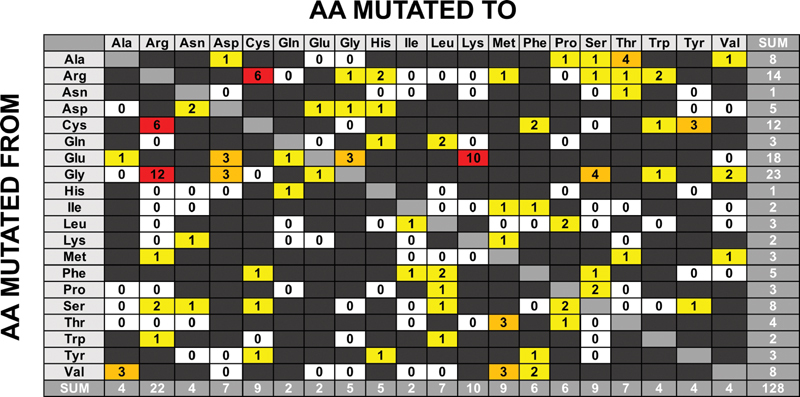
Substitution grid representing 128 missense variants in the *F10* gene. The grid illustrates the number of variants of each defined amino acid change. All substitutions shown are the result of a single nucleotide change. Any substitutions that require more than a single nucleotide change are shown in dark gray. Silent variants are excluded from the grid and shown in pale gray. The white boxes represent possible substitutions that do not occur in the *F10* gene. The yellow boxes represent substitutions that occur once or twice, the orange boxes represent substitutions that occur three or four times and the red boxes represent substitutions that occur five or more times.

Substitution analysis was performed on 128 FX missense variants to predict the damaging effects of each amino acid change. All the substitutions involved single nucleotide changes; none appeared in the greyed boxes of Fig. 5 . As illustrated in Fig. 5 , the total count for each missense change is shown, with Gly, Glu, Arg, and Cys being the most commonly mutated residues (23, 18, 14, and 12 cases, respectively). For these four residues, a change involving a charged residue is often observed, such as Gly > Arg, Glu > Lys, and Cys > Arg. Such substitutions are readily seen to be protein damaging. Of interest, the Gly residues (23) were the most commonly affected. This concurs with Gly being the smallest residue and thus its replacement with a bulkier residue is likely to cause protein structural rearrangements. Charged residues are also more often varied than others; Glu (18) and Arg (14) are the second and third most commonly substituted residues. The removal of a charged group in a protein can often destabilize its structure. Cys residues are the fourth most commonly mutated residue. The formation of Cys–Cys disulphide bridges is key to protein folding stabilization, so the loss of one bridge can be disruptive to the overall three-dimensional structure.

### Phenotype and Accessibility Analysis of Factor X Missense Variants

The merged FXa crystal structure (Methods) resulted in a full-length FXa model that permitted structural analyses of the variants. When subjected to the Ramachandran stereochemical analysis, the 1XKA crystal structure (used for the EGF-1, EGF-2, and SP domains) revealed 92.8% of all residues to be in the most favored conformational regions, with a further 6.6% of residues in the additional allowed regions. The 1P0S crystal structure (used for the GLA domain) revealed that 90.3% of all residues were in the most favored conformational regions, with a further 8.2% of residues residing in additional allowed regions. The 1XKA structure showed two Ramachandran outliers (Leu113 and Arg126), while the 1P0S structure showed seven (Leu131, Gln144, Asn145, Asp159, Asn160, Gln356, and Glu369). The merged FXa model revealed that 99.4% of all residues were in favored regions with 91.8% of these residing in the most favored regions. The only two Ramachandran outliers found in the merged model were Leu113 and Arg126, present also in the EGF domains in the 1XKA crystal structure. 24 25 26 The merged FXa structure was concluded to be of high enough quality for an accurate structural assessment of the FX variants.

**Fig. 6:**
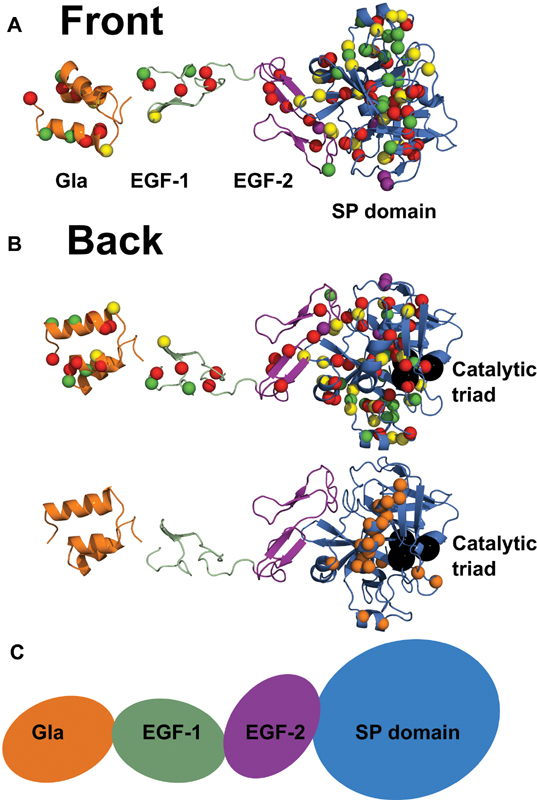
Structural view of the distribution of variants within FXa. ( **A** and **B** ) illustrate the front and back views (rotated by 180°) of the distribution of 113 missense variants and 4 polymorphisms within mature factor X (FX). The merged FXa crystal structure is shown in ribbon format, with the Cα positions of the variants and polymorphisms highlighted as spheres. The variant locations are colored according to their phenotype. Type-I (CRM − ) variants are in red, type-II (CRM + ) variants in green, and those with unknown phenotype in yellow. The nondisease associated polymorphisms are depicted in purple. The catalytic triad His276-Asp322-Ser419 (HGVS numbering) is shown in ( **B** ) in the SP domain as black spheres. In ( **B** ) also, orange spheres denote the residues Arg313, Lys316, Arg346, Arg 387, Lys391, Lys460, and Arg469 (HGVS numbering) correspond to a heparin binding site in FX (chymotrypsin numbering Arg93, Lys96, Arg125, Arg165, Lys169, Lys236, and Arg240). These heparin binding residues together with Asp413-Asp418 and Val455-Arg469 (HGVS numbering) are involved with Factor Va binding (chymotrypsin numbering Asp185-Asp189 and Val231-Arg245). The ribbon colors correspond to those used on our web site *https://www.factorx-db.org* . EGF, epidermal growth factor; GLA, γ-carboxyglutamic acid; SP, serine protease.( **C** ) Schematic representation of the four domains in FX. The four domains are aligned and depicted in the same orientation and colors as in the ribbons in ( **A** and **B** ) above.

The merged FXa structure was used to show the distribution of 113 missense variants and 4 nondisease associated polymorphisms in mature FXa ( Figs. 6A and B ). The GLA domain contained three α-helices, A1–3, and a single π-helix P1. The EGF-1 and EGF-2 domains were primarily β-sheet structures, with EGF-1 forming two β-strands A-B and EGF-2 forming four β-strands A-D. The SP domain contained 13 β-strands A-M, as well as three minor α-helices, three 3 _10_ -helices, and one π-helix ( Fig. 4 ). A schematic representation of the FXa domain structure showed that the four FXa domains were in an extended arrangement ( Fig. 6C ). In the merged FXa structure, 186 out of 488 residues were assigned accessibilities of 0 or 1, meaning that their sidechains were buried, and a further 186 residues were assigned accessibilities of 2 or more, indicating that their sidechains were solvent exposed ( Fig. 4 ). The remaining 116 residues were not present in the FXa protein structure and thus were unavailable for accessibility analysis. These 116 residues included the 40-residue pre–pro leader peptide, the 52 amino acid AP removed on FX activation, and several others including those at the end of the SP domain. Of the 127 variants with defined accessibilities, several of which occurred at the same location in the FX sequence, 49 were classified as surface exposed with accessibilities of 2 or more and 78 were classified as buried with accessibilities of 0 or 1 ( Fig. 4 ).

When observing the full-length FXa structure, the ratio of type-II to -I variants stood at 0.35, while this ratio increased to 0.44 when looking solely at the SP domain. Given the catalytic function of the SP domain, unsurprisingly type-II variants occurred more frequently in the SP domain compared with the rest of FXa. The clustering of the type-II variants (green) was visible in the SP domain when mapped onto the FX model ( Fig. 6A ). Interestingly, of the 17 type-II point variants in the SP domain, three had surface accessibilities of 2 or more, indicating sidechain exposure, while the remaining 14 were classified as buried. Such findings indicated that, regardless of surface exposure, the SP variants were able to impede native FXa function without disrupting the overall FXa structure, resulting in type-II defects. In contrast, the type-II variants in the GLA, EGF-1, and EGF-2 domains were all at exposed locations, suggesting that these may affect interdomain or intermolecular interactions in the functioning of FX. The predominance of type-I variants in the GLA, EGF-1, and EGF-2 domains suggested that these domains were more important in maintaining an extended FXa structure rather than having a functional role.

**Fig. 7:**
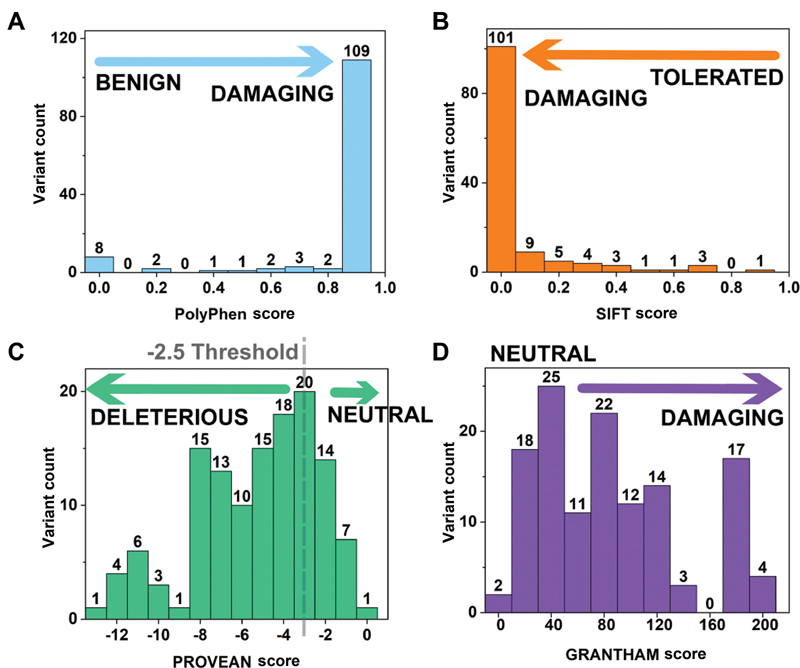
Substitution analysis of 128 missense variants in the *F10* gene. Substitution analyses predict the damaging effects of substitution variants. ( **A** ) Graphical distribution of variants determined by their PolyPhen-2 score; ( **B** ) Graphical distribution of variants determined by their the Sorting Intolerant From Tolerant (SIFT) score; ( **C** ) Graphical distribution of variants determined by their PROVEAN score. The PROVEAN threshold used was -2.5; ( **D** ) Graphical distribution of variants determined by their Grantham's score.

The observation of a missense FX variant in the gene of a patient is not necessarily causative of a bleeding disorder. To assist interpretations, the FX web site offered guidelines to clarify whether a given variant is likely to disturb the overall protein structure and function. The PolyPhen-2 algorithm predicted that 109 (85%) of the 128 variants were damaging, with scores of 0.9 to 1.0 ( Fig. 7A ). The SIFT analysis predicted that 110 (86%) of variants were damaging with scores of 0.0 to 0.2 ( Fig. 7B ). This outcome indicated that most FX variants were causative for FX deficiency as expected. The PROVEAN and Grantham analyses demonstrated less clear trends, although the majority of variants showed damaging scores ( Fig. 7C and D ). The interactive FX database provides variant-specific PolyPhen-2, SIFT, PROVEAN, and Grantham scores for the 128 missense variants, thus providing an easy-to-use clinical support tool to clarify the significance of a variant ( Fig. 2 ). The significance of the four scores for a given variant is clarified from Fig. 7 . A high damaging score would highlight that the substitution in question is likely to explain FX deficiency in the patient.

**Fig. 8:**
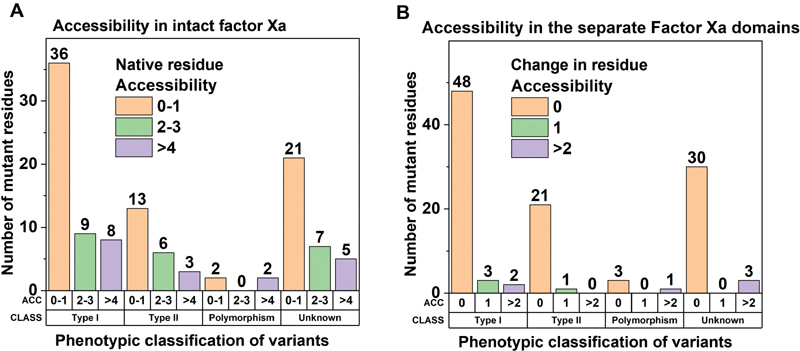
Accessibility analyses of 108 FX missense variants and four polymorphisms in the factor X (FX) protein structure. ( **A** ) FX variants in the FXa structure are grouped by phenotypic classification (CLASS). The variants are further subdivided according to the native residue accessibility (ACC) of the intact protein. Accessibility was determined using the Definition of Secondary Structure of Proteins (DSSP) and is explained in detail in the Methods section. Accessibilities of 0 or 1 indicate sidechain burial and values >1 indicate sidechain exposure to solvent. ( **B** ) FX variants are again grouped by phenotypic classification (CLASS) and accessibility (ACC). Here, accessibility refers to the change in residue accessibility when the intact FX protein is separated into its four distinct domains, namely, the γ-carboxyglutamic acid (GLA), epidermal growth factor (EGF)-1, EGF-2, and serine protease (SP) domains.

To further examine the relationship between FX phenotypes and surface accessibilities, the residue surface accessibilities were displayed as a function of phenotypes for (1) the intact FXa protein and (2) the individually separated FXa domains ( Fig. 8 ). Notably, a high 68% of type-I variants (36 of 53) showed accessibilities of 0 or 1, highlighting their predisposition to be buried within the intact FXa protein structure ( Fig. 8A . In contrast, type-II variants and polymorphisms appeared in both the surface-exposed and buried regions of the FXa structure, with no clear preference for either location. Many variant residues were of unknown phenotype; however, interestingly the majority of these showed low accessibilities. Following the separation of the four domain structures in FX, the resulting changes in surface accessibility compared with the intact protein enabled the identification of residues that made interdomain contacts. An even higher proportion of 91% of type-I variants (48 of 53) was located to residues that showed no accessibility change after domain separation ( Fig. 8B ). The same outcome was also seen for type-II variants and unassigned phenotypes. The predominance of variants at buried locations within FX showed that small perturbations in the FXa structure, through the introduction of variants that lead to slight changes in surface accessibility, are sufficient to impair the protein and lead to disease states.

**Fig. 9:**
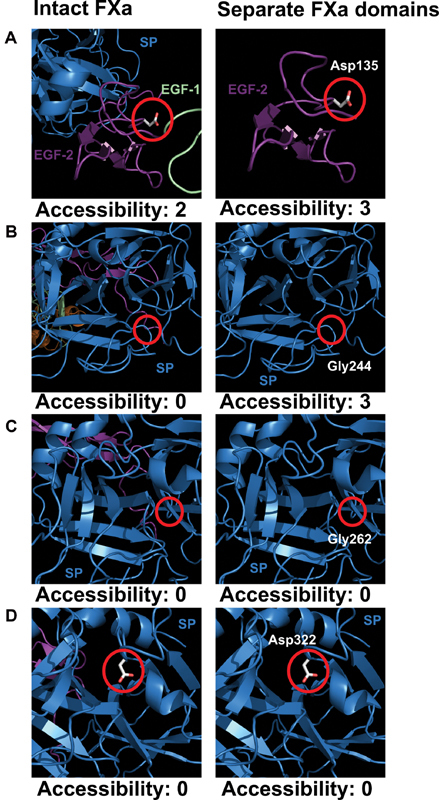
Molecular graphic representation of four selected variants in the FXa protein structure. The views are taken from the unmodified FXa structural model (Methods). The left-hand panel highlights the residue of interest and its calculated solvent accessibility in the intact FXa protein. The right-hand side panel shows the same residue in its respective isolated domain, with its corresponding accessibility. ( **A** – **D** ) highlight the residues Asp135, Gly244, Gly262 ( Fig. 2 ) and Asp322 (catalytic triad) respectively. Accessibility calculations are detailed in the Methods section. The domain coloring corresponds to that in Fig. 5 . All residue numbering is given in HGVS format.

To illustrate the utility of the web site, four individual variant residues with low surface accessibilities were visually highlighted on the FXa model ( Fig. 9 ), namely, (1) Asp135Glu in EGF-2 (type I, accessibility 2), (2) Gly244Arg/Glu in SP (type I, accessibility 0), (3) Gly262Asp in SP (type I, accessibility 0), and (4) Asp322Asn in SP (type II, accessibility 0). The separation of the domain structures alters the accessibility of Asp135 from 2 to 3, and that of Gly244 from 0 to 3. These views explain the visual context of residue substitutions by three-dimensional inspections of the native FXa structure. It should be noted that these changes (particularly to buried residues) may be causing local structural changes in the protein. For the first variant, the replacement of an Asp with a Glu residue seen at the EGF-1 and EGF-2 interface was sufficient to destabilize the FXa protein folding, presumably by altering the interdomain interface to form a less stable one. For the second and third variant residues, the replacement of a buried Gly residue by a much bulkier charged Arg, Glu, or Asp residue was likely to destabilize the protein folding of the SP domain, resulting in the observed type-I phenotype. The third variant is exemplified in Fig. 2 . The fourth example is the substitution of Asp322 by Asn322 within the catalytic triad His276-Asp322-Ser419. The change impacts only on FXa function, not on the overall protein structure, thus explaining why this is reported as a type-II defect. Missense variants at such locations have perturbed these inter- and intradomain interactions, disrupting the native FXa structure and resulting in protein degradation or dysfunction. Thus, the web site provides molecular explanations for the relative abundance of type-I and -II variants in FX in terms of small but significant disruptions to the intricately folded domain structure, even though the FXa domain structure is an extended one and relatively solvent exposed.

## Discussion

The compilation of 180 genetic variants for FX in this study represents to our knowledge, the first interactive web database for FX that summarizes documented patient records, their genetic variants, and protein structural analyses, all assembled as one convenient resource. The new FX web site represents the continuation of our other related interactive web sites for FVII, FVIII, FIX, and FXI ( *https://www.factorvii.org* , *https://www.factorviii-db.org* , *https://www.factorix.org* , and *https://www.factorxi.org* ). 20 21 22 23 37 For FX, this web site will significantly improve the quality of variant analyses and will lead to further insights into the occurrence of FX disease states. Most notably, we show that these variants are observed across the entire FXa protein structure ( Fig. 6 ), and that accessibility changes in the amino acid packing arrangement in the folded FXa structure by residue substitution is a major cause of FX deficiency ( Fig. 8 ). Accessibility changes may be a good predictor for the changes associated with a variant. The disruption scores from Polyphen-2, SIFT, PROVEAN, and the Grantham offer useful insight into the impact of the variants and will provide the clinician with guidelines on the significance of a variant. Such an analysis of the FX variants has resulted from three main advances as follows: (1) the availability of sufficient literature on FX genetic variants as the result of the increasing use of patient genotyping ( Fig. 1 ); (2) the availability of a merged FXa crystal structure of a sufficient high quality for analyses (Methods); and (3) our increasing expertise in developing interactive databases related to hemophilia. 23

Many multidomain plasma proteins present extended domain arrangements. Examples include the three-dimensional structures for FXa, activated FVII, and activated FIX, all three of which have the same four domains termed GLA-EGF1-EGF2-SP. Other plasma proteins present more compact domain arrangements such as that of FXI. 23 Both FX and FXI present variants that are distributed throughout their protein structures, implying that the extended or compact nature of their domain structures do not influence variant distributions. However, where the phenotypes are known, FVII, FIX, and FX variants show a higher proportion of type-II phenotypes which are associated with functional defects, rather than type-I phenotypes which correspond to misfolding defects. 20 21 22 23 37 This outcome is as expected given that FVII, FIX, and FX, all have extended domain arrangements. FXI shows a higher proportion of type-I defects, and this outcome is attributed to its compact domain arrangement which is more easily damaged by disease-associated variants. Heparin and Factor Va exosites are known for FX 38 and both are shown on the SP domain of Fig. 6B , together with the catalytic triad. Of interest is that many type-II variants in the SP domain (green) are spatially close to these exosites.

## Conclusion

Our interactive FX database serves as a useful resource for clinicians and scientists to diagnose FXI deficiency and provide insight into the effect of a variant. Database technology becomes required in the light of large increases in the known genetic variants in FX, when a simple spreadsheet listing of mutations is no longer adequate to monitor these. The web site layout is designed to present genetic and structural information on FX as two distinct but parallel themes, similar to that for our original FVII, FVIII, FIX, and FXI databases. 20 21 22 23 37 This is illustrated using genetic and structural outputs for the commonly-reported Gly262Asp variant for which 20-patient records exist ( Fig. 2 ). On the left, further insight into the conservation of Gly262 is obtained from the AA alignments tab which shows Gly262 aligned with six other mammalian species. This alignment reveals that the Gly262 residue is conserved in only three other sequences and therefore may be able to accommodate a bulkier sidechain. On the right, the structural analysis shows that Gly262 is a buried residue on a β-strand, and the JMol viewer shows that this is located inside the SP domain. Further research into FX, including experimental studies, will be key to a better understanding of the relationship between FX deficiency and disease severity.

## Notes

**What Is Known on This Topic**

1. Many Factor X (FX) genetic variants have been published in the literature yet, there is no interactive database which compiles both variant and patient information and offers sequence and structural variant analysis.
2. A total of 136 FXa crystal structures are available on the Protein Databank web site ( *https://www.rcsb.org* ), yet there are no available structural models that show all four FXa domains.

**What This Paper Adds**

1. Our new interactive FX database enables sequence and structural analysis of *F10* genetic variants identified prior to April 2021 and serves as a literature pool of an up-to-date FX resource.
2. Information regarding 180 unique FX variants from 427 case reports is presented in our interactive database of which 174 variants are disease-associated and 6 are nondisease-associated polymorphisms.
3. We present a full-length structural model of FXa created from two previously published FXa crystal structures, and present guidelines to assess the likely impact of newly reported missense variants.
